# Dynamic occupancy models for analyzing species' range dynamics across large geographic scales

**DOI:** 10.1002/ece3.858

**Published:** 2013-11-07

**Authors:** Florent Bled, James D Nichols, Res Altwegg

**Affiliations:** 1 South African National Biodiversity InstituteP/Bag X7, Claremont, 7735, South Africa; 2 Animal Demography Unit, Departments of Biological Sciences and Statistical Sciences, University of Cape TownRondebosch, 7701, South Africa; 3 Patuxent Wildlife Research Center, US Geological SurveyLaurel, Maryland, 20708

**Keywords:** Autologistic model, big data, conservation biogeography, hierarchical model, spatially correlated random effects

## Abstract

Large-scale biodiversity data are needed to predict species' responses to global change and to address basic questions in macroecology. While such data are increasingly becoming available, their analysis is challenging because of the typically large heterogeneity in spatial sampling intensity and the need to account for observation processes. Two further challenges are accounting for spatial effects that are not explained by covariates, and drawing inference on dynamics at these large spatial scales. We developed dynamic occupancy models to analyze large-scale atlas data. In addition to occupancy, these models estimate local colonization and persistence probabilities. We accounted for spatial autocorrelation using conditional autoregressive models and autologistic models. We fitted the models to detection/nondetection data collected on a quarter-degree grid across southern Africa during two atlas projects, using the hadeda ibis (*Bostrychia hagedash*) as an example. The model accurately reproduced the range expansion between the first (SABAP1: 1987–1992) and second (SABAP2: 2007–2012) Southern African Bird Atlas Project into the drier parts of interior South Africa. Grid cells occupied during SABAP1 generally remained occupied, but colonization of unoccupied grid cells was strongly dependent on the number of occupied grid cells in the neighborhood. The detection probability strongly varied across space due to variation in effort, observer identity, seasonality, and unexplained spatial effects. We present a flexible hierarchical approach for analyzing grid-based atlas data using dynamical occupancy models. Our model is similar to a species' distribution model obtained using generalized additive models but has a number of advantages. Our model accounts for the heterogeneous sampling process, spatial correlation, and perhaps most importantly, allows us to examine dynamic aspects of species ranges.

## Introduction

Some of the most pressing problems in nature conservation (e.g., biodiversity loss, climate change-induced range shifts) play out at large geographic scales (Root et al. 2003, Gaston 2003, Parmesan 2006), and addressing them requires biodiversity data collected across large areas (Jetz et al. 2011). This type of data set is becoming more and more available and is making it possible for key ecological questions to be addressed in new ways (Hampton et al. 2013). For example, one development is the newly emerging field of conservation biogeography (Richardson and Whittaker 2010), which applies macroecological concepts to conservation (Kerr et al. 2007).

However, drawing robust inference from large-scale ecological data is challenging. Data sets that span wide geographic areas are typically heterogeneous because it is difficult to collect those data in a standardized way. Researchers increasingly rely on citizen scientists to contribute to data collection (Greenwood 2007). Citizen science allows researchers to obtain detailed data sets across large spatial scales, and rigorous data collection protocols are often employed. However, the analysis of those data sets is challenging, because detection probabilities tend to vary spatially, for example due to variable sampling effort, and because the large number of contributors is bound to lead to variable levels of skill.

All observational data reflect both the underlying biological process and the observation process (Williams et al. 2002). Even with relatively standardized sampling protocols, population estimates can be imprecise or biased simply because of the partial nature of the information gathered through the observation process (Kéry 2011). Therefore, this process should be explicitly accounted for in the analyses (Altwegg et al. 2008; Kéry et al. 2010). Another complication with the analysis of large-scale data sets is that they usually exhibit spatial autocorrelation (Latimer et al. 2006). This can sometimes lead to biased inference if ignored (Dormann et al. 2007; Beale et al. 2008), especially in the case of uneven spatial sampling or if accuracy at a fine scale is desired. Spatial relationships are clearly important when analyzing dynamic processes, such as colonization and extinction (Bled et al. 2011).

There is therefore a need for robust methods to analyze large-scale data sets as an underpinning for research in macroecology and biogeography, including conservation biogeography. Ideally, methods should offer a flexible way to account for the observation process and spatially correlated effects. These methods should also allow for an analysis of the dynamics underlying large-scale biodiversity patterns, such as local extinction and colonization, and permit inferences about environmental covariate effects.

Dynamic occupancy models (MacKenzie et al. 2003, 2006) offer a framework for analyzing large-scale species distribution data while accounting for the observation process (Kéry et al. 2010). Occupancy models are designed to separate the underlying biological process responsible for species distribution, from the observation process. The sampling protocol requires that spatial units be sampled repeatedly within a short enough time span to ensure that a species is either always present or always absent within a sampling season. Based on this closure assumption, one detection establishes a site as occupied, and other detections and nondetections provide information about detection probability conditional on presence. The closure assumption can be violated in various ways. If the species colonizes or goes extinct from sites during the period over which closure is assumed, estimates of detection probabilities may be biased, leading to biased estimates of occupancy probabilities (Rota et al. 2009). Species may be temporarily absent from sites, for example if the home ranges of individuals are larger than the spatial sampling unit or if species use habitats seasonally. In this case, occupancy can be interpreted as space use (MacKenzie et al. 2006) and estimates are unbiased when space use is random.

The closure assumption is relaxed in dynamic occupancy models (MacKenzie et al. 2003). Dynamic occupancy models (MacKenzie et al. 2003) assume closure over sampling seasons and allow for extinction and colonization between seasons. The appeal of dynamic occupancy models for species distribution data is that they include parameters that determine the dynamics of species distributions, allowing researchers to determine what drives these dynamics (Altwegg et al. 2008).

Here, we develop a dynamic hierarchical occupancy model to analyze bird atlas data collected across South Africa, Lesotho, and Swaziland during two atlas projects (Harrison et al. 1997, 2008). This model has to encompass the spatial autocorrelation that occurs at such a scale, dynamic processes occurring at different timescales (both between and within the two atlas projects), and the specificities of each project's sampling designs. Moreover, this model has to be general in order to be applied to species with different life-history traits. In order to illustrate the use of the model, we apply it to study the range dynamics of the hadeda ibis (*Bostrychia hagedash*), a species that has naturally expanded its range across southern Africa over the past 100 years (Macdonald et al. 1986).

## Methods

### Data

To monitor the distributions of bird species, two atlas projects were conducted across southern Africa. Data for the first Southern African Bird Atlas Project (SABAP1) were collected mostly between 1987 and 1992, whereas field work for SABAP2 started in June 2007 and is still ongoing in 2013 (Harrison et al. 1997; Harebottle et al. 2007). Both projects employed a similar protocol: volunteers collected checklists of all bird species they saw during a birding session within predetermined regular grid cells that span the whole region. For SABAP1, these were quarter-degree grid cells, whereas for SABAP2, they were 5′ × 5′ grid cells. To compare the data between the two projects, we pooled SABAP2 data across the nine grid cells that correspond to a quarter-degree cell. Even though 2894 (SABAP1) and 985 (SABAP2) observers contributed to data collection, 90% of the data were collected by 25% (SABAP1) and 27% (SABAP2) of the observers. The large majority of checklists were collected by intensely birding for a few hours, even though volunteers were allowed to add species to their lists for up to 30 days in SABAP1 and up to 5 days in SABAP2. The protocol for SABAP2 further imposed a minimum of 2 hours of intense birding and asked birders to note the hour of intense birding during which a species was first seen. Species encountered after the intense birding but within the 5 days limit were recorded as such. Both atlases asked birders to note each species only once, regardless of how many individuals were seen. Our analysis included the 2025 quarter-degree grid cells covering South Africa, Swaziland, and Lesotho (see Figs S1–S3). Multiple checklists were collected per year for many grid cells. Both projects employed a rigorous vetting process to identify possible misidentifications and other errors (see Harrison et al. 1997 and Harebottle et al. 2007 for details).

We developed a model to estimate range dynamics from these data. As environmental covariates on initial occupancy, we used the proportion of area occupied by the relevant vegetation types in each grid cell, using data from Mucina and Rutherford (2006). The eight biomes/categories we considered were the savanna biome, Albany thicket biome, forests biome, fynbos biome, Indian ocean coastal belt, grassland biome, Nama-karoo biome, and an “others” category (grouping desert, succulent karoo biomes, azonal vegetation, and waterbodies). Hadedas need trees for breeding and open, relatively moist habitat for feeding (Duckworth et al. 2010). We therefore expected that occupancy would differ between forests, savannah, fynbos, and the more arid karoo biomes.

### Model

**Figure 1:**
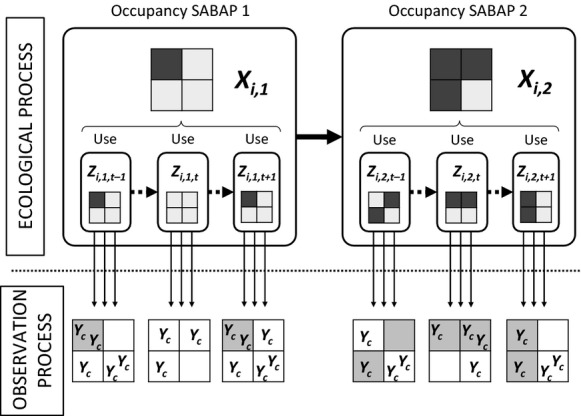
Model diagram representing the relationship between the three hierarchical levels of occupancy *X*_*i,s*_, use *Z*_*i,s,t*_*,* and observation *Y*_*c*_. The four-cell grid represents a simple spatial lattice where the species of interest can either be present (dark cells) or absent (white cells) within the time period of interest (SABAP or year depending on the temporal scale). The plain horizontal arrow represents the dynamic processes of cell persistence and colonization between SABAPs. Dashed horizontal arrows indicate dynamic processes of exploitation and appropriation within each SABAP. As illustrated, the number of checklists collected varies between cells and years.

We modeled the observed occupancy *Y*_*c*_ (*Y*_*c*_ = 1 if the species is detected and 0 if not) on checklist *c* for the species of interest using a hierarchical approach. In this hierarchical model, we considered three levels reflecting two ecological processes at two timescales and the observation process. First, we modeled the distribution at the scale of each SABAP (i.e., occupancy). Then, the yearly occupancy (referred hereafter as use) within each SABAP is modeled conditionally on the occupancy at the SABAP level. Finally, the detection/nondetection data are modeled conditionally on the yearly use. A general graphical representation of this model is presented in Figure 1.

#### First level: Occupancy during SABAP1 and SABAP2

We are particularly interested in the species' distribution over each SABAP and how this distribution changed between the two projects. Occupancy is then defined as the species' distribution within the atlas region during one SABAP, that is, all the grid cells where the species might be found, even though they are not guaranteed to actually be present in any given year (or indeed, with a small probability, at all during an atlas period). Therefore, if we consider *i* = 1, 2,…, *N* spatial units (i.e., grid cells), the first ecological process level described occupancy *X*_*i,s*_ (*X*_*i,s*_ = 1 if occupied, 0 if not occupied) in cell *i*, during SABAP_*s*_. We model occupancy *X*_*i,s*_ in cell *i*, during SABAP_*s*_ by a Bernoulli distribution with parameter *q*_*i,s*_ as:


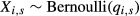


The Bernoulli parameters for SABAP1 and SABAP2 are modeled differently. While we modeled occupancy probability during SABAP1 directly, occupancy during SABAP2 was derived from previous occupancy status and a dynamic process of colonizations and extinctions (see below). Occupancy during SABAP1 was estimated using generalized additive models (GAM) to account for the habitat structure based on the vegetation data and spatially structured and unstructured random effects.


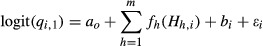


where *a*_o_ is an intercept, *f*_*h*_(*H*_*h*,*i*_) are smooth functions linking occupancy probabilities to *H*_*h,i*_ habitat covariates (i.e., percentage of cell *i* covered by habitat/biome *h*). The smooth functions *f*_*h*_ () were modeled using spline functions with two knots as described in Crainiceanu et al. (2005). Finally *b*_*i*_ and *ɛ*_*i*_ are the spatially structured and unstructured random effects for cell *i*.

The spatially correlated random effects *b*_*i*_ are expressed as a CAR model where the spatial effect of the cell *i* is based on contiguous grid cells, those cells that share a common boundary or corner with cell *i*. Specifically, we use an intrinsic version of the CAR model analogous to that proposed by Besag et al. (1991). The Gaussian CAR model for the spatially correlated random effect *b*_*i*_ can then be defined as


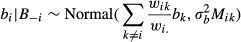


where *B* is the vector [*b*_*1*_,…, *b*_*N*_], and *B*_*−i*_ the corresponding vector that omits *b*_*i*_. Connectivity between cell *i* and cell *k* is represented by element *w*_*ik*_ (*w*_*ik*_ = 1 if cells are neighbors, 0 otherwise). *M*_*ik*_ is a *N* × *N* diagonal matrix (where *N* denotes the total number of cells) with elements *M*_*ii*_ proportional to the conditional variance of *b*_*i*_|*B*_*−i*_, 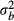 is the conditional variance parameter. In the intrinsic model, we set *M*_*ii*_ = 1/*n*_*i*_, where *n*_*i*_ is the number of neighbors of cell *i*. Essentially, *b*_*i*_ has a normal distribution with conditional mean given by the average of the spatially correlated random effects of its neighbors. The conditional variance is inversely proportional to the number of neighbors of *b*_*i*_.

Occupancy during SABAP2 resulted from processes of persistence (a previously occupied cell may stay occupied) and colonization (a previously unoccupied cell may become occupied). Occupancy probability of a cell during SABAP2 was then defined as the result of a first-order Markov process conditional on cell occupancy state during SABAP1, as in the dynamic occupancy models presented by MacKenzie et al. (2006), Royle and Kéry (2007), and Bled et al. (2011):


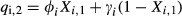


where *φ*_*i*_ and *γ*_*i*_ are persistence and colonization probabilities for cell *i* between SABAP1 and SABAP2. Those probabilities are then defined as:


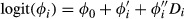



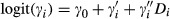


with *φ*_0_ and *γ*_0_ are intercepts, 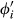 and *γ*′_*i*_ random cell effects, and *φ*″_*i*_ and *γ*″_*i*_ slopes for the response of persistence and colonization probabilities to neighborhood occupancy *D*_*i*_. *D*_*i*_ is a covariate defined as the proportion of first-order neighboring cells to cell *i* (i.e., grid cells that share a common boundary or corner with cell *i*) occupied during SABAP1. A cell that has a large number of occupied neighbors is more likely to stay occupied (rescue effect of Brown and Kodric-Brown 1977) or to become colonized (e.g., Hanski 1998). This is an autologistic model (Bled et al. 2011; Yackulic et al. 2012).

#### Second level: Use within each SABAP

We view occupancy as a description of the species' range within the study area, even though a grid cell may not be used by the species continuously during SABAP_*s*_. Our model therefore had a second ecological process describing use *Z*_*i,s,t*_ of cell *i*, during year *t* of SABAP_*s*_. Introducing this dynamic component allowed us to relax the closure assumption so that we only require closure within each year but not throughout the full atlas periods. We modeled use *Z*_*i,s,t*_ in cell *i*, during year *t* of SABAP_*s*_ by a Bernoulli distribution with parameter *μ*_*i,s,t*_ and conditionally on occupancy *X*_*i,s*_ such as:


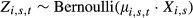


If cell *i* is not occupied during SABAP_*s*_, that is, *X*_*i,s*_ = 0, then use *Z*_*i,s,t*_ is also equal to 0. If cell *i* is occupied during SABAP_*s*_, then the use probability is equal to *μ*_*i,s,t*_.

Initial cell use probabilities for SABAP1 and SABAP2, that is, *t* = 1, were assumed to be iid Bernouilli random variables, conditioned on cell occupancy status *X*_*i,s*_ and with *μ*_*i,s,1*_ having a prior distribution uniform between 0 and 1. In subsequent periods, the use probabilities *μ*_*i,s,t*_ were defined conditionally on the previous year's use status *Z*_*i,s,t*−1_ (as well as occupancy status *X*_*i,s*_) and dynamics parameters such as:


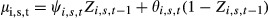


where the dynamics of the use status within each SABAP were modeled by two parameters: exploitation probability *ψ*_*i*,*s*,*t*_ (or its complement, cell-specific abandonment, 1 − *ψ*_*i*,*s*,*t*_), and appropriation probability *θ*_*i*,*s*,*t*_. Exploitation probability *ψ*_*i*,*s*,*t*_ corresponds to the probability of continued use of cell *i* between year *t* and year *t +* 1 during SABAP_*s*_; it is similar to persistence probability at the occupancy level. Appropriation probability *θ*_*i*,*s*,*t*_ corresponds to the probability of cell *i* being used in year *t + 1*, after not having been used in year *t* and is similar to colonization probability at the occupancy level. Exploitation probability and appropriation probability are furthermore modeled as:


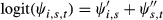



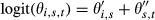


where 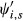 and *θ*′_*i*,*s*_ are random cell effects, and *ψ*″_*s*_,_*t*_ and *θ*″_*s*,*t*_ are random year effects, for exploitation and appropriation probabilities, respectively.

#### Third level: Observation process

Finally, we modeled observed occupancy *Y*_*c*_ for checklist *c* (i.e., in year *t* during SABAP_*s*_ for cell *i*, by observer *k*) by a Bernoulli distribution conditional on use *Z*_*i,s,t*_ with detection probability *p*_*c*_ such as:


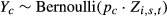


Since sampling design protocols were slightly different between SABAP1 and SABAP2, we had to model detection probability differently for the two SABAPs. For the modeling of detection probability for SABAP1, we defined detection probability at the checklist level *p*_*c*_ as


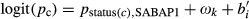


where *p*_status(c),SABAP1_ is the intercept describing the mean detection probability for the species depending on its seasonal breeding status at the time when checklist *c* was collected. In our example, we distinguish between June to November versus December to May, which corresponds to courtship, and breeding versus nonbreeding seasons for most resident birds in our region. The breeding status can be thought of as a general seasonal effect. Here, seasonal breeding status defines periods of homogeneous detection probabilities that could vary throughout a year, depending on the species' biology. For hadedas, we expected detectability to be higher when they are breeding than when they are not breeding. Parameters *ω*_*k*_ and *b′*_*i*_ correspond to random observer effects for observer *k* and spatially structured random effects for cell *i*, respectively. The spatially structured random effects for cell *i* are defined similarly as presented above for occupancy, using a CAR model, and were introduced to account for variation in detection probability caused primarily by spatial variation in abundance.

For SABAP2, we had more information about factors that could have affected detection probability. We knew (1) whether the species was detected during the initial period of intense birding, (2) and if so, during which hour of this initial period. Therefore, detection probability at the checklist level for SABAP2 was defined as


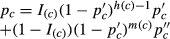


where *I*_*(c)*_ is an indicator function indicating if species detection for checklist *c* occurred during the initial period of intense birding (*I*_(*c*)_ = 1), or not (*I*_(*c*)_ =0), *h*(*c*) is hour of first detection, *m*(*c*) is the number of hours spent birding intensely for checklist *c*, and *p′*_*c*_ is the hourly detection probability during the period of intense birding. The probability of detecting the species anytime after the initial period of intense birding is denoted as *p′′*_*c*_. These probabilities were defined as


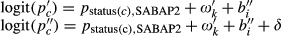


where *p*_status(c),SABAP2_ is the intercept describing the mean hourly detection probability for the species depending on its seasonal breeding status at the time when checklist *c* was collected, *ω′*_*k*_ is a random observer effect, *b”*_*i*_ corresponds to a spatially structured random effect, and *δ* is the difference in detection probability between the period of intense birding and subsequent less intense birding. These definitions of *p′*_*c*_ and *p″*_*c*_ are similar to the definition of the global detection probability of SABAP1, except that *p′*_*c*_ is an hourly detection probability and *p″*_*c*_ is the detection probability over the whole undefined period of time following the initial intense birding period of *m*_*(c)*_ hours.

#### Implementation

We implemented the model using program WinBUGS (Lunn et al. 2000). We ran three chains using noninformative priors, for 50,000 iterations after a 150,000 iteration burn-in period. The WinBUGS code for our model is provided in Appendix S1 of the Supporting Information.

### Example

We modeled the dynamics of the southern African range of the hadeda ibis (*Bostrychia hagedash*). Hadedas are relatively conspicuous birds because of their loud and characteristic calls and tendency to forage in open spaces. They do not resemble any other species that occurs in the region. Hadedas are undergoing a range expansion in our study area at least since the early 1900s (Macdonald et al. 1986), probably due to land use change (Duckworth et al. 2010). The species is detected over most of South Africa and seems to have extended its range between the two projects (Fig. S3).

## Results

**Figure 2:**
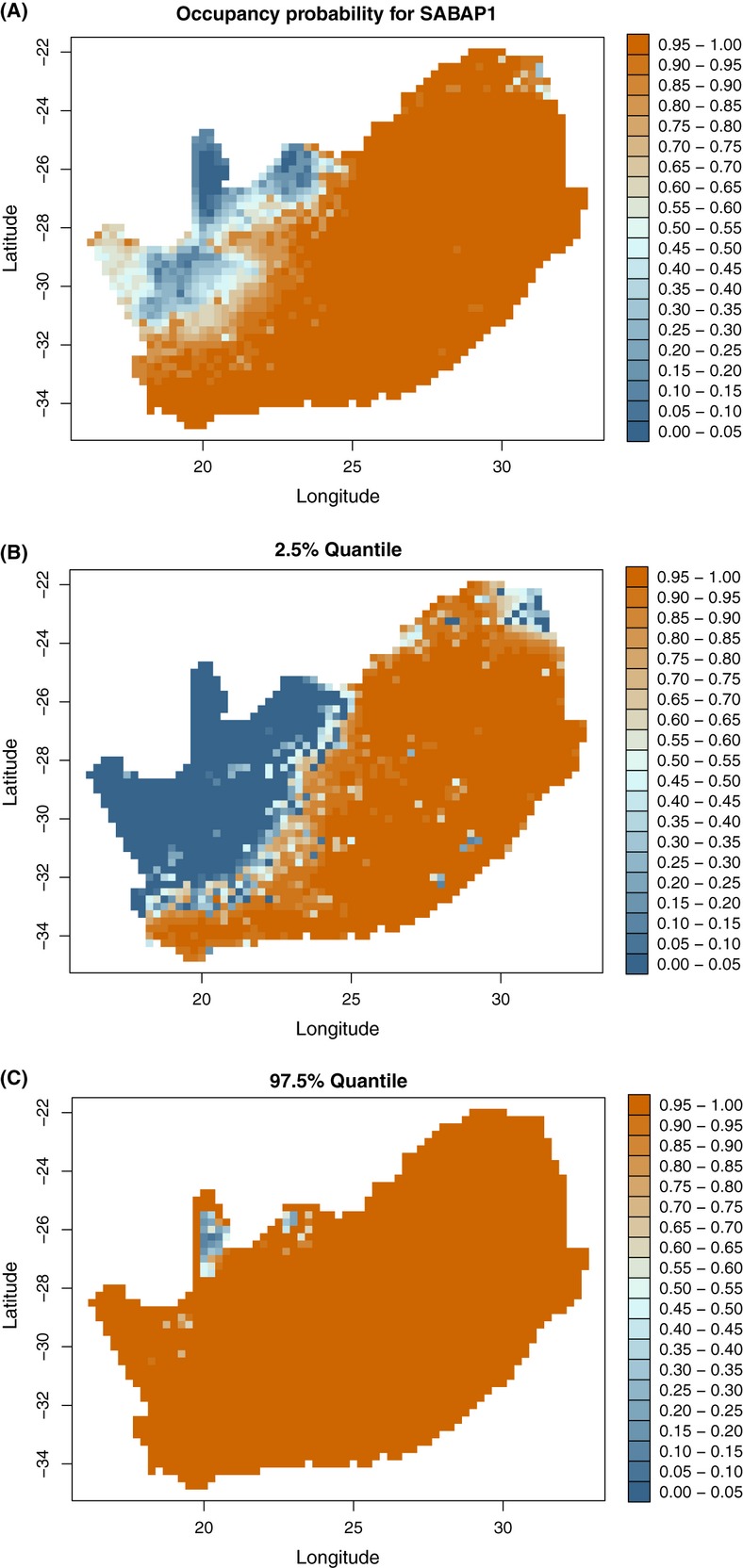
(A) Estimated mean occupancy probability of the hadeda ibis (*Bostrychia hagedash*) based on checklist data collected during the first Southern African Bird Atlas Project (SABAP1, 1987–1992). Panels (B) and (C) show the 2.5th and 97.5th quantiles of the posterior distribution.

**Figure 3:**
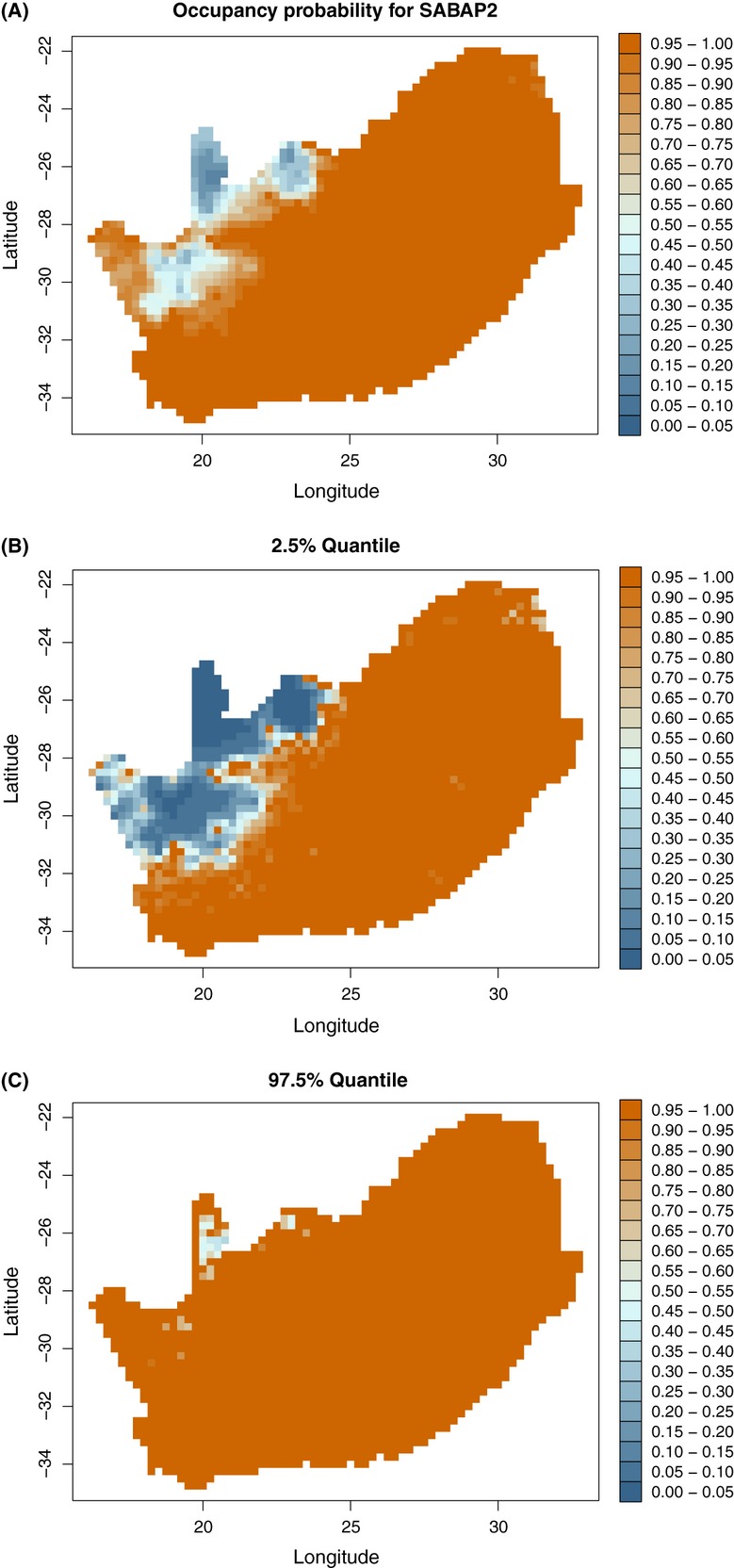
(A) Estimated mean occupancy probability of the hadeda ibis (*Bostrychia hagedash*) based on checklist data collected during the second Southern African Bird Atlas Project (SABAP2, 2007–2012). Panels (B) and (C) show the 2.5th and 97.5th quantiles of the posterior distribution.

*Occupancy dynamics between SABAP 1 and 2* – The hadeda was widely present over South Africa during SABAP1 (Fig. 2) and SABAP2 (Fig. 3) with occupancy probabilities over 0.8 for most of South Africa. Only in the northwestern part of the country were the occupancy probabilities lower (under 0.5 during SABAP1). The northwestern part of South Africa is also a relatively remote area where data collection effort has been low (Figs S1 and S2). This led to a high uncertainty in the occupancy probabilities in this area (Fig. 2).

**Figure 4:**
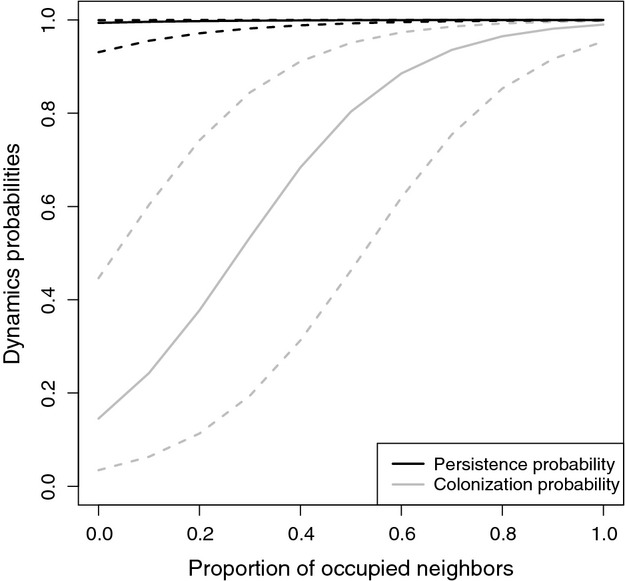
The estimated persistence probability (probability of an occupied grid cell to remain occupied) and colonization probability (probability of an unoccupied grid cell to become occupied) in relation to the number of occupied neighbors. (Corresponding 95% credible intervals indicated by the dashed lines.)

Occupancy increased from SABAP1 to SABAP2 (Fig. 3), and estimated occupancy probabilities were high throughout the study area for SABAP2. This reflects the observed range expansion well, even though the uncertainty in occupancy probabilities was still high for the northwestern part of the country. Overall, the proportion of occupied cells between SABAP 1 and 2 increased by 8.2% [95% credible interval 4.5; 11.0%]. This was the result of high persistence and colonization probabilities. Persistence probability was overall homogenous over South Africa (between 0.9 and 1, Fig. S4). Colonization probability showed a spatial structure with a low probability in the north of South Africa and in areas mainly dominated by deserts (Fig. S5). Persistence and colonization probabilities were positively correlated with the number of occupied surrounding grid cells (Fig. 4), even though the persistence probability was always high. Little local extinction seems to have happened during the course of our study, which agrees with the observation that this species is generally increasing in South Africa (Duckworth et al. 2010). The colonization probability was low (<0.2) for cells surrounded by unoccupied neighbors (*D* = 0%), but increased quickly with increasing neighborhood occupancy.

**Figure 5:**
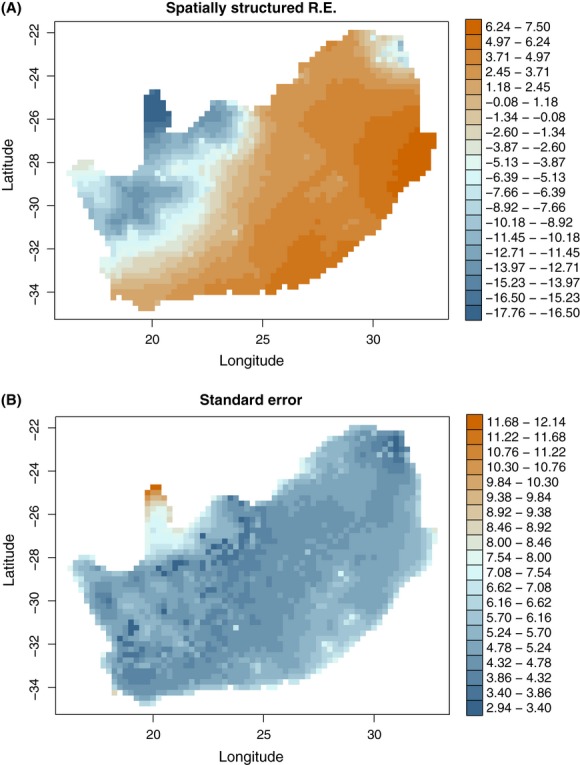
(A) Spatially structured random effect (CAR component) for occupancy probability of hadedas during the first Southern African Bird Atlas Project (SABAP1), and (B) standard error.

The spatially structured random effects for occupancy during SABAP1 showed a gradient going from southeast to northwest (Fig. 5), while the unstructured random effect showed no particular spatial pattern (Fig. S6). This indicates that the spatial autocorrelation in occupancy was effectively captured by the spatial covariates (habitat) and the CAR component.

**Figure 6:**
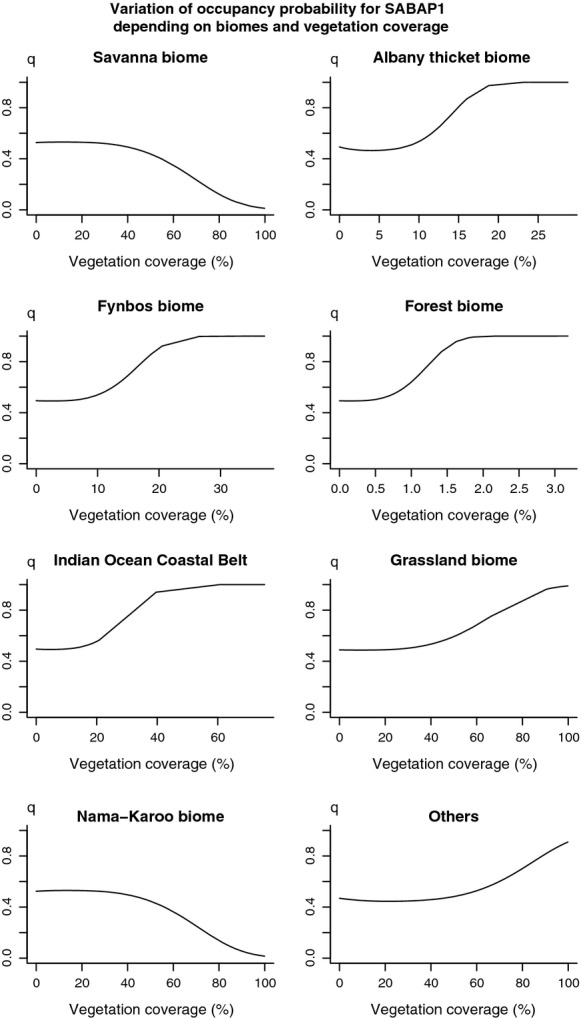
Estimated relationship between hadeda occupancy probability during SABAP1 and habitat covariates. The habitat covariates were the percentage of each cell's area that was covered by each vegetation types.

Hadedas were more likely to occupy grid cells during SABAP1 that had a higher percentage covered by Albany thicket, fynbos, forest, Indian Ocean coastal belt, and grassland biomes (Fig. 6). As expected, occupancy probability was negatively correlated with the presence of savannah and Nama-karoo biomes.

**Figure 7:**
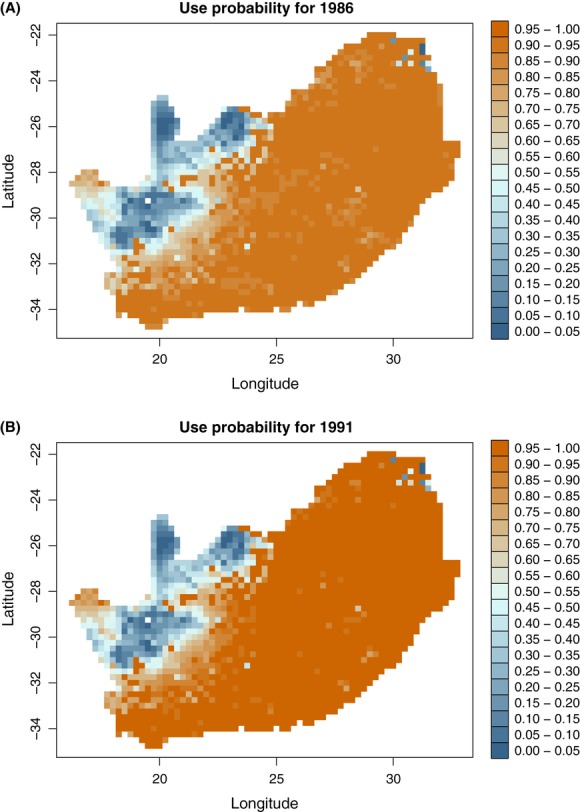
The probability of an occupied grid cell being used by hadedas in a particular year. We show the estimates for the years (A) 1986 and (B) 1991 as examples.

*Use within SABAPs* – Use within each SABAP stayed rather constant, even though slight variation in use probabilities among years indicated that the species presence in each grid cell varied during each atlas project. In 1986, the core of the species' range in this region (southwest of South Africa) had an average use probability between 0.8 and 0.85 (Fig. 7, upper panel). Five years later, this probability increased to 0.90–0.95 (Fig. 7, lower panel). The inclusion of a hierarchical level relaxed the closure assumption that one would have had to make by treating each atlas period as a single season. Modeling use allowed us to detect slight year-to-year variation in presence while providing a good representation of the occupancy over the full duration of each project.

*Observation process* – Because of the slightly different sampling designs, our estimates of the detection probability is per checklist for SABAP1 and per hour of birding for SABAP2. These two detection probabilities cannot be directly compared. However, we found that the detection probabilities during the breeding season were higher than detection probabilities during the nonbreeding season during both projects. For SABAP1, the detection probability at the checklist level was higher by 0.39 [0.34; 0.45], on the logit scale, during the breeding season compared with the nonbreeding season. For SABAP2, the hourly detection probability increased by 0.06 [0.02; 0.09] on the logit scale during the breeding season over the nonbreeding season.

During SABAP1, there was a clear spatial pattern in detection probability with relatively higher detection probabilities in the southwestern part of the region (Fig. S7). During SABAP2, this spatial component was less pronounced (Fig. S8). This could be due to the difference in sampling design (and therefore modeling). The standard error maps of the spatially structured random effects for detection probability reflect patterns in sampling intensity for each SABAP (Figs S1 and S2). There was considerable variation in detection probabilities among observers in both atlas projects (Fig. S9). Such variation is expected when data are collected by a large and potentially heterogeneous group of observers.

## Discussion

We developed an occupancy model for analyzing biodiversity data that is conceptually similar to a GAM-based species' distribution model, which is currently a popular tool for analyzing large-scale occurrence data (Elith and Leathwick 2009). In addition, however, the dynamic occupancy model allowed us to examine the range dynamics of hadedas across the subcontinent, while accounting for the observation process. We believe that accounting for the observation process is particularly important in large-scale data sets where sampling effort and detection probabilities almost necessarily vary spatially. Among the less heterogeneous data sets are the ones collected by coordinated atlas projects.

Atlas projects typically aim at mapping species occurrence across large areas. A common design for conducting atlases is to divide the area into a regular grid and attempt to collect data for all grid cells over a limited time. This general protocol was also employed for two bird atlases in southern Africa, SABAP1 and 2. In the case of the SABAP, observers were asked to collect checklists, leading to repeated detection/nondetection data for the >700 bird species found on the subcontinent.

The SABAP data have a number of properties that are typical for this type of data. Most importantly, these are uneven spatial coverage (see Figs S1 and S2), variable effort per checklist, and a large number of observers with potentially heterogeneous skills (see Fig. S9). These properties form the observation process that makes the raw data a distorted representation of the true processes we want to study. Separating the observation process from the biological process generally requires either repeated observations of the process at least in some portion of the grid cells or else potentially restrictive assumptions about covariate relationships determining occupancy and detection parameters (Lele et al. 2012). Site occupancy models (MacKenzie et al. 2002, 2006) are one statistical approach designed for this situation.

To relax the closure assumption made by occupancy models, we added a dynamic component within the seasons, which in our case were the main atlas periods. Adding this extra level allows a focal species to be temporarily absent, and therefore not recordable, from grid cells that it occupies in the longer term. We call this level “use”, following MacKenzie et al. (2006). We found a slight increase in use within SABAP1 that was in line with the expansion of the species' range between the atlases (see Fig. 7).

At the spatial resolution of our data, we expected occupancy dynamics to be more clearly manifested over the 15-year time step between the two projects compared with yearly time steps. We therefore selected an approach that focuses on the dynamics of range expansion over a 15-year time frame. One consequence of defining the atlas period as a season and modeling yearly use within season is that a grid cell could potentially be estimated to be occupied but never used, which rarely happened in our case. Alternatively, one could define occupancy as the probability of a grid cell being used at least for 1 year within each season. Models based on this approach would not condition use (*Z*_*i,s,t*_) on seasonal occupancy, but would instead treat the latter as a derived parameter. Under such an approach, occupancy probability would equal zero when a cell has not been used at all, but it would be harder to model occupancy directly as a function of covariates. Both modeling approaches are reasonable, and we selected the one that we thought to be most consistent with our objectives that focused on range dynamics between the two SABAP periods and broad-scale occupancy within each period.

Another general property of grid-based sampling designs is that neighboring grid cells may not be independent, even after accounting for possible shared habitat covariates. We found that modeling the spatial effects was important in our case. We used conditional autoregressive models (CAR, Besag et al. 1991) to account for residual spatial autocorrelation in occupancy during SABAP1 and detection probabilities in both atlases. The observation process also appeared to be spatially autocorrelated, and this could be due to variation in abundance affecting detection probabilities. Another approach to deal with abundance-induced spatial heterogeneity in detection would have been to utilize detection information to infer abundance (Royle and Nichols 2003). Modeling spatial autocorrelation in occupancy models is currently a field of active development (Johnson et al. 2013). Additional covariates could explain part of the residual spatial autocorrelation. Covariates could also be incorporated at the use level (Z_i,s,t_) and for modeling persistence and colonization parameters where they could provide valuable information about drivers of use and occupancy dynamics.

We modeled spatial dependencies in persistence and colonization probabilities using autologistic models (Bled et al. 2011; Yackulic et al. 2012); that is, these probabilities depended on the number of neighboring grid cells that were occupied during SABAP1. Autologistic models may fail if too many grid cells are not sampled at all. Where it works, however, in our opinion, this model makes biological sense (i.e., provides a mechanistic model) because unoccupied grid cells are more likely to be colonized from nearby occupied sites than from sites further away (Hanski 1998; Clobert et al. 2001). Likewise, persistence may be increased in neighborhoods with high occupancy because of the rescue effect (Brown and Kodric-Brown 1977). Modeling range dynamics in this way can give important information on how fast species may colonize suitable habitat, an important parameter for projecting both species range shifts under climate change and invasion speed (Neubert and Caswell 2000; Altwegg et al. 2013). A big limitation of current species' distribution models is that they cannot realistically account for dispersal limitation (Midgley et al. 2006).

Citizens have become an important partner in scientific projects that require data collected across a large spatial scale (Greenwood 2007). This is an especially gratifying collaboration, because this gives researchers a direct way to connect with the general population and increases awareness for big challenges such as biodiversity loss and climate change. However, there is often a conflict between making the data collection protocol stringent enough to allow for robust analysis, and making it simple enough for observers to enjoy participating and be able to adhere to the protocol. The big advantage of using grid- and checklist based protocols is that they provide repeated detection/nondetection data. Repetition would be difficult to achieve with point-based protocols, where often not much about the observation process is known.

Macroecological questions, by their very nature, require data from the typically large geographic scale of species ranges. Historically, macroecological questions have been addressed primarily by identifying patterns (e.g., in species distribution) and then trying to infer underlying processes from these patterns (e.g., Brown 1995). Because most patterns can potentially be explained by numerous underlying processes, these inferences have been widely challenged and are characterized by substantial uncertainty (Strong et al. 1984; Gaston and Blackburn 1999). An alternative approach to inference about dynamic processes is to study these processes directly (e.g., see discussion in MacKenzie et al. 2006). That was our approach in this modeling effort, and we note that it can easily be adapted to other atlas data sets. Certainly, important macroecological conservation questions about changes in species distributions in response to land use change and climate change can be readily addressed using this approach.

## Supporting Information

**Figure S1.** Sampling effort during the first Southern African Bird Atlas Project (SABAP1): logarithm of the number of checklists plus one per quarter-degree grid cell.

**Figure S2.** Sampling effort during the second Southern African Bird Atlas Project (SABAP2): logarithm of the number of checklists plus one per quarter-degree grid cell.

**Figure S3.** Map showing detections of hadeda ibis (*Bostrychia hagedash*) during the two Southern African Bird Atlas Projects (SABAP1 and 2).

**Figure S4.** The probability of hadedas to persist in occupied grid cells between 1992 and 2007, and 2.5% and 97.5% quantiles.

**Figure S5.** The probability of hadedas colonizing unoccupied grid cells in southern Africa between 1992 and 2007, and 2.5% and 97.5% quantiles.

**Figure S6.** Unstructured random effects for hadeda occupancy probability during the first Southern African Bird Atlas Project (SABAP1), and standard error.

**Figure S7.** Spatially structured random effects for detection probabilities of hadedas during the first Southern African Bird Atlas Project (SABAP1).

**Figure S8.** Spatially structured random effects for detection probabilities of hadedas during the second Southern African Bird Atlas Project (SABAP2).

**Figure S9.** Posterior distribution for the standard deviation in detection probabilities of hadedas among observers during the first Southern African Bird Atlas Project (SABAP1, panel A) and during the second Southern African Bird Atlas Project (SABAP1, panel B).

**Appendix S1.** BUGS code used to fit the model.
