# Supplementary material for: Dynamic occupancy models for analyzing species' range dynamics across large geographic scales
Source: Ecol Evol. 2013 Nov 7;3(15):4896–909. doi: 10.1002/ece3.858 (PMC3892356; doi:10.1002/ece3.858)
Supplement: Supplementary file 1 [file ece30003-4896-SD1.pdf]

Supporting Information

For: Bled, Nichols & Altwegg 2013: Dynamic occupancy models for analysing species' range dynamics across large geographic scales. Ecology & Evolution.

Figure S1. Sampling effort during the first Southern African Bird Atlas Project (SABAP1): logarithm of the number of checklists plus one per quarter degree grid cell.

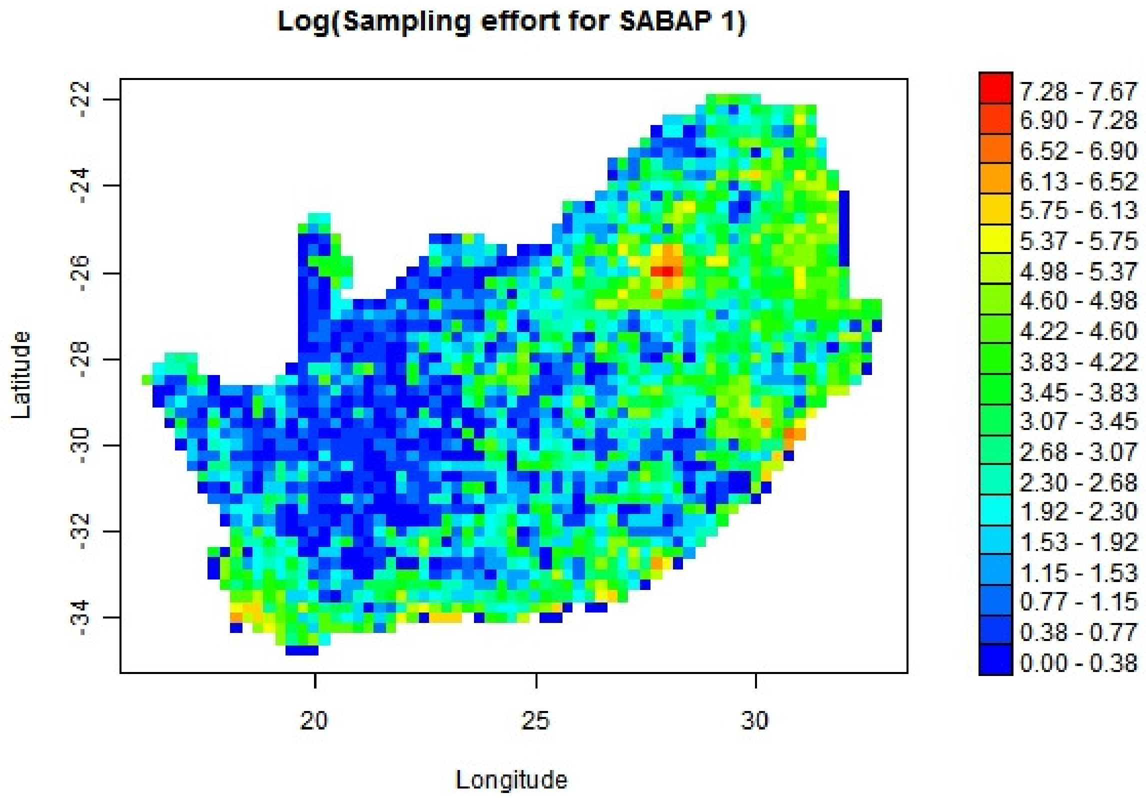

Figure S2. Sampling effort in during the second Southern African Bird Atlas Project (SABAP2): logarithm of the number of checklists plus one per quarter degree grid cell.

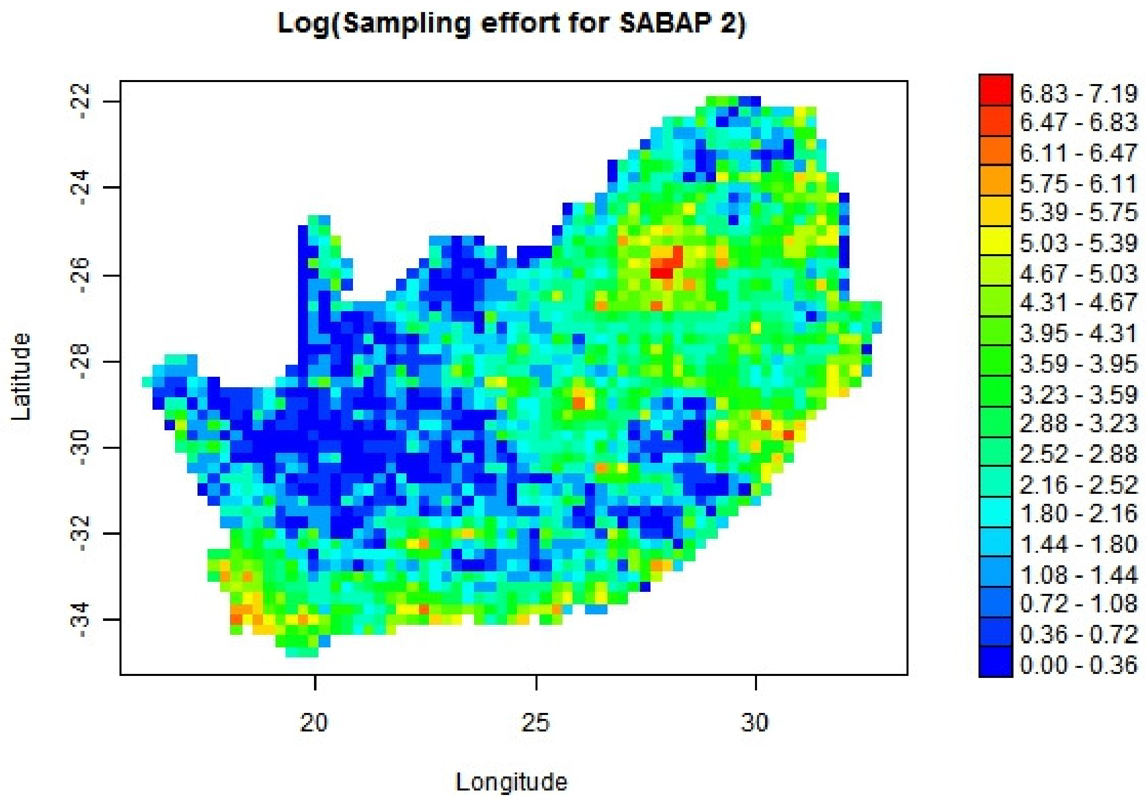

Figure S3. Map showing detections of hadeda ibis (*Bostrychia hagedash*) during the two Southern African Bird Atlas Projects (SABAP1 and 2). The map shows in which quarter-degree grid cell the species was seen during both projects (dark green), SABAP1 only (grey), and SABAP2 only (red). Hadedas were not recorded in the light green cells.

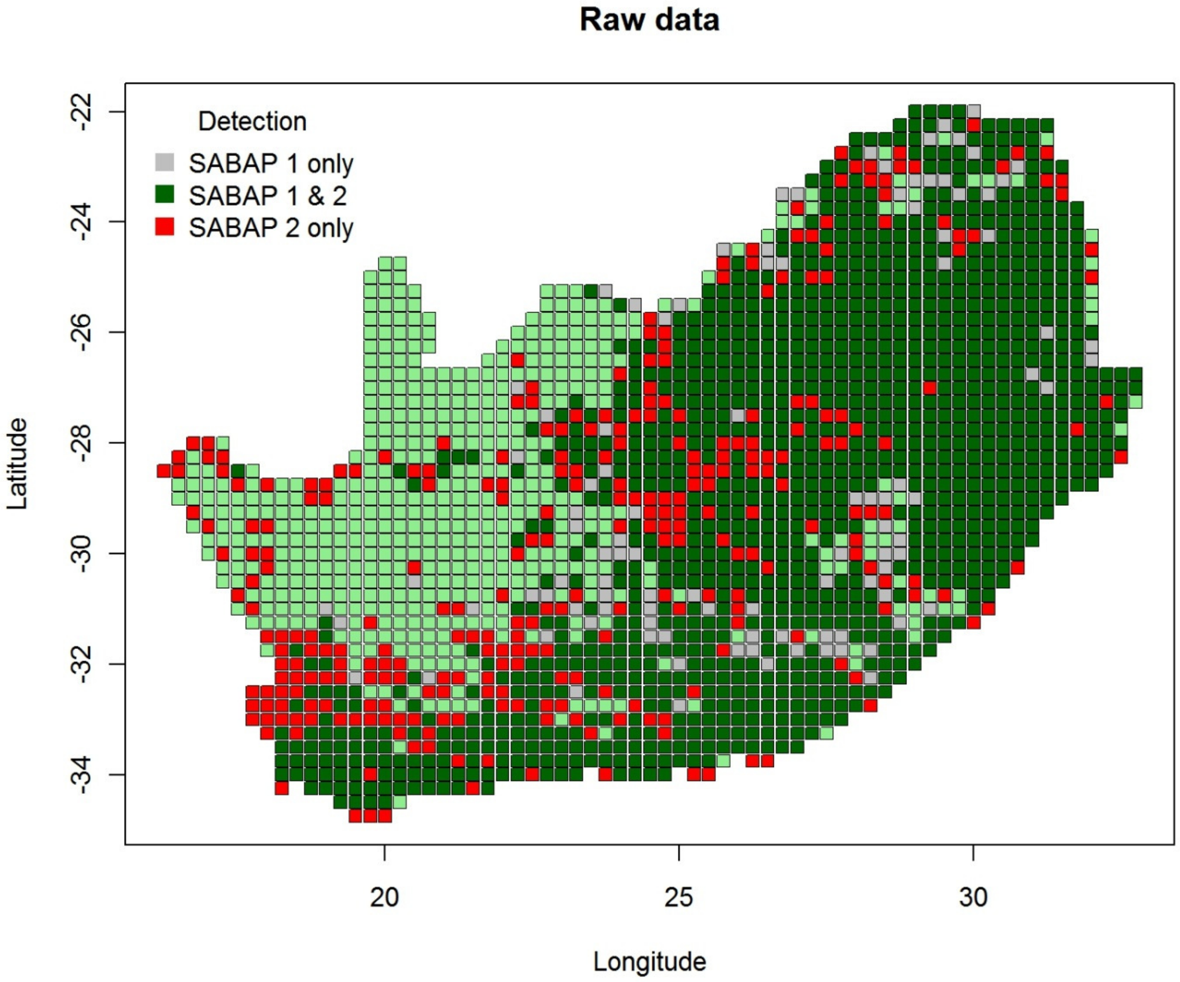

Figure S4. The probability of hadedas to persist in occupied grid cells between 1992 and 2007, ,

and 2.5% and 97.5% quantiles .

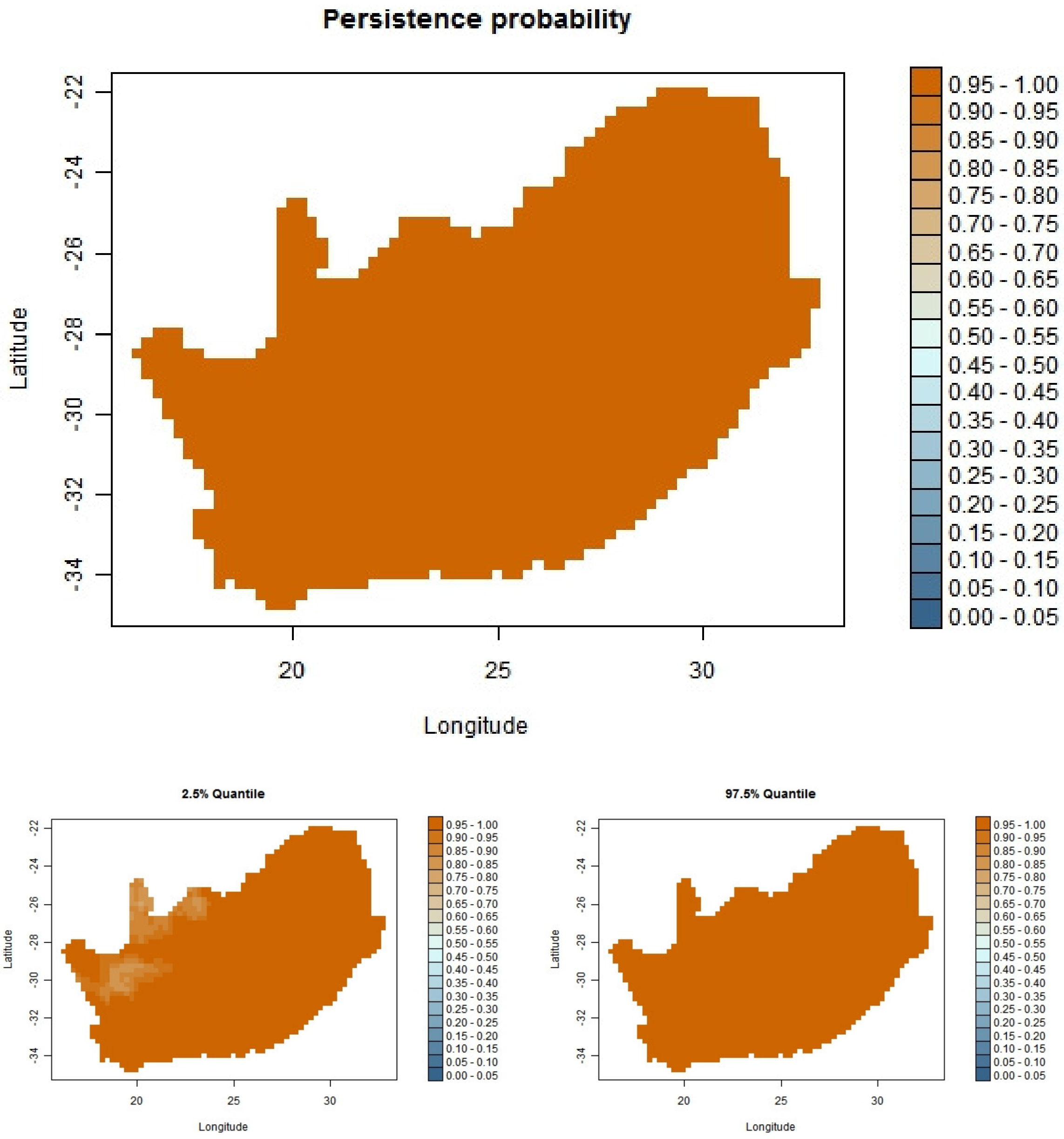

Figure S5. The probability of hadedas colonising unoccupied grid cells in southern Africa between 1992 and 2007, and 2.5% and 97.5% quantiles.

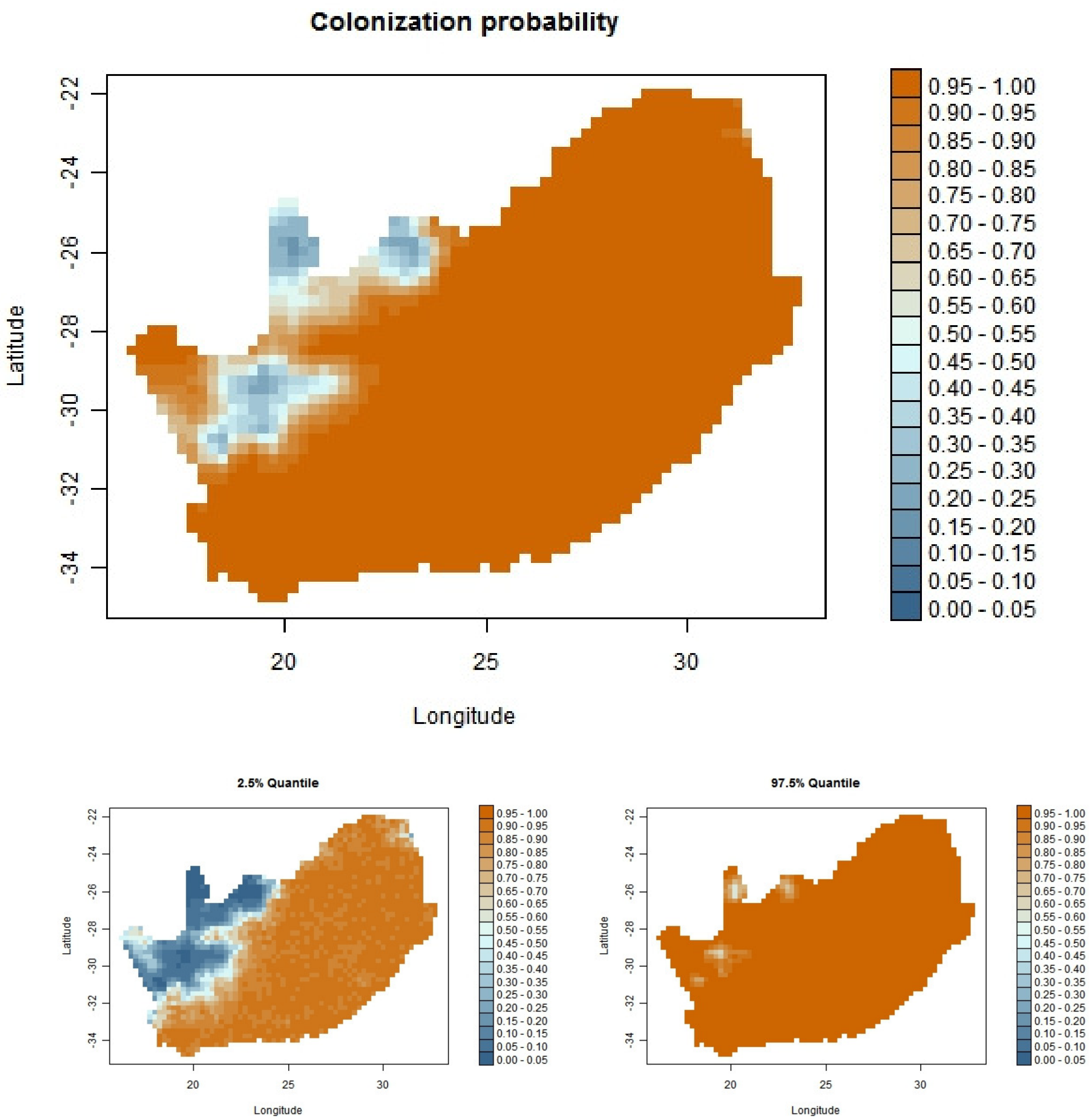

Figure S6. Unstructured random effects for hadeda occupancy probability during the first Southern African Bird Atlas Project (SABAP1), and standard error. The purpose of these random effects was to account for remaining variation in occupancy that was not explained by the habitat covariates and the spatially structured random effects.

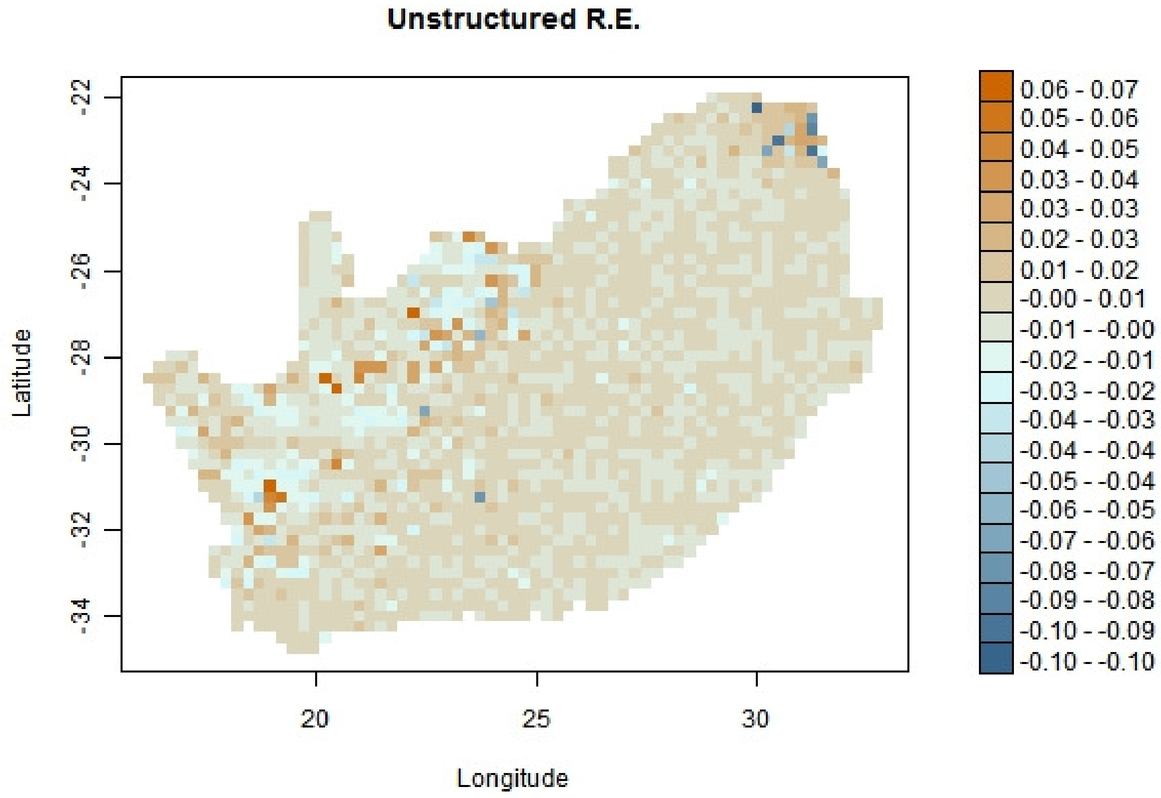

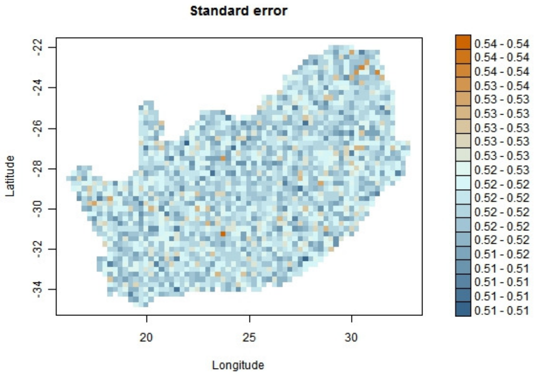

Figure S7. Spatially structured random effects for detection probabilities of hadedas during the first Southern African Bird Atlas Project (SABAP1).

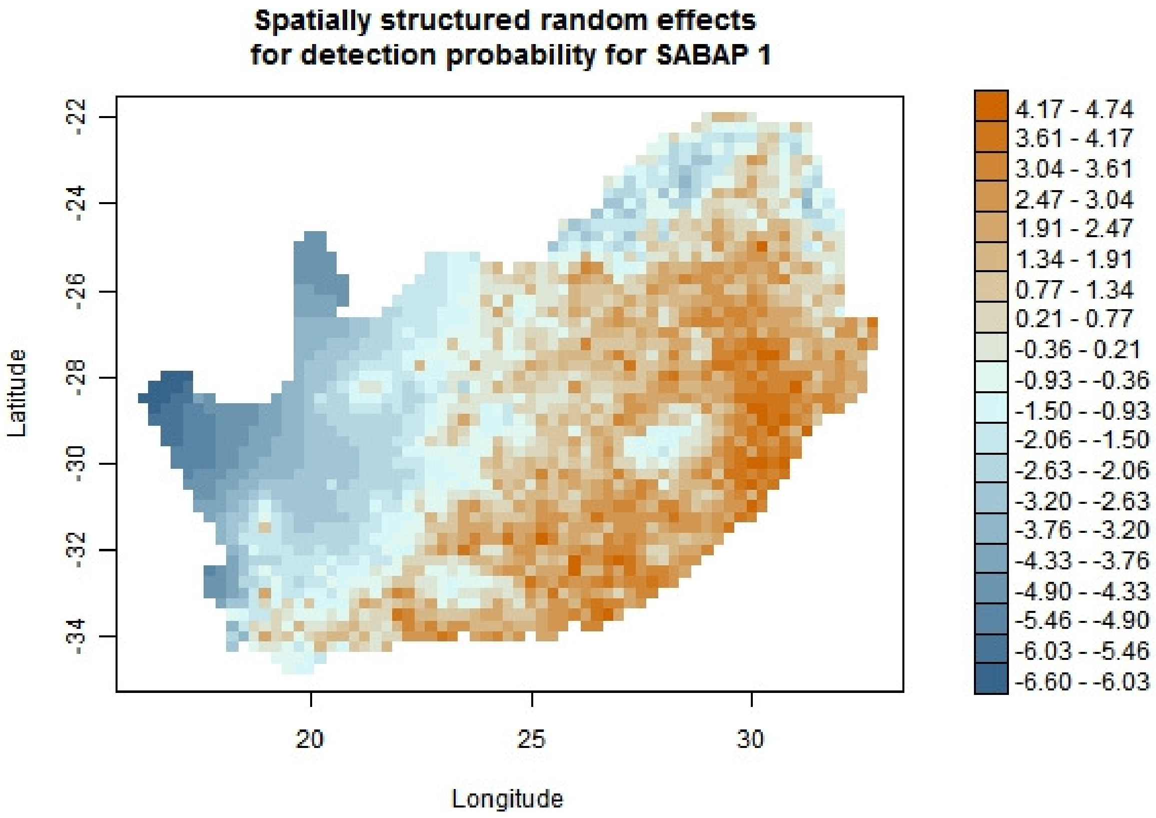

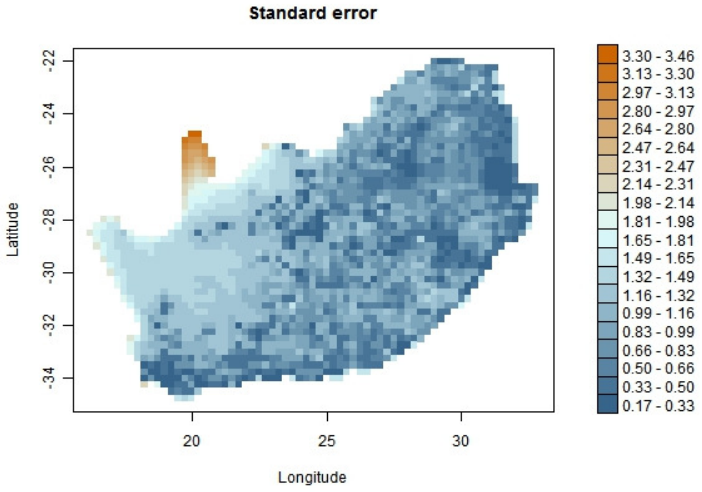

Figure S8. Spatially structured random effects for detection probabilities of hadedas during the second Southern African Bird Atlas Project (SABAP2).

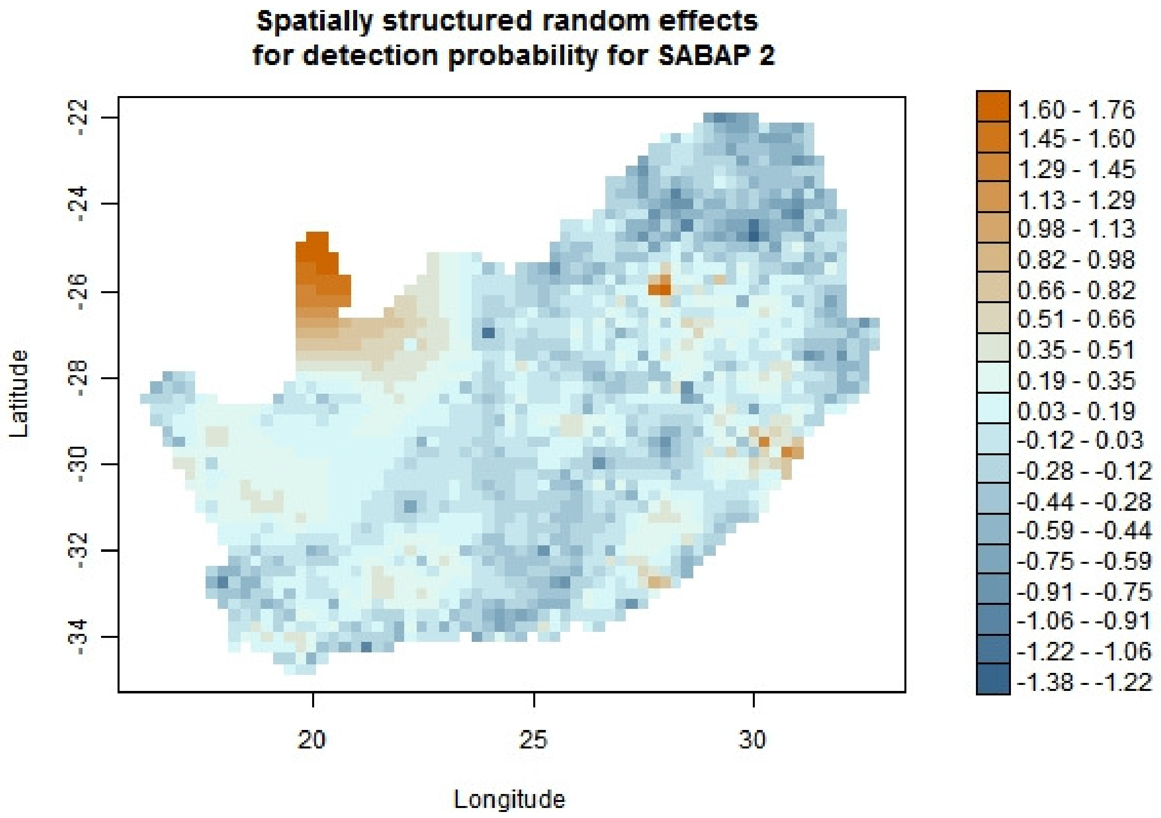

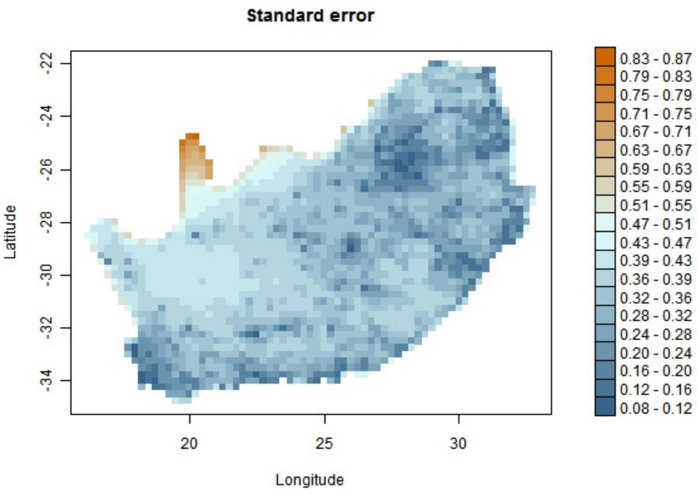

Figure S9. Posterior distribution for the standard deviation in detection probabilities of hadedas among observers during the first Southern African Bird Atlas Project (SABAP1, panel A) and during the second Southern African Bird Atlas Project (SABAP1, panel B).

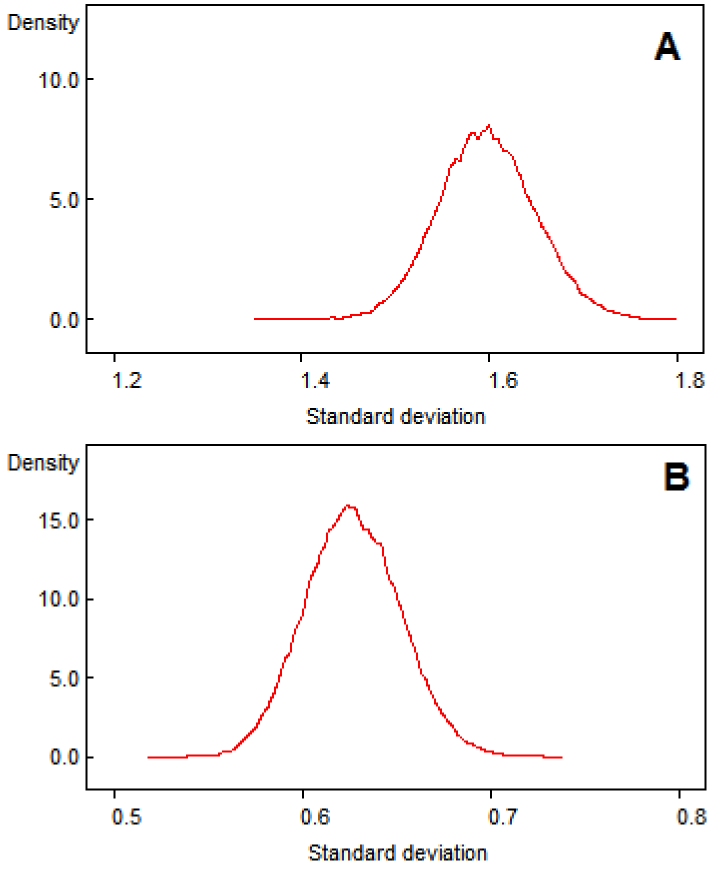

Appendix S1. BUGS code used to fit the model.

```
### Note:
###      On a computer with 12GB of RAM and 2 3.47GHZ Intel Zeon processors,
###      the analysis took a little bit over a week for the Hadeda ibis.

model {

 ##############################################################
 #
 #          ### DATA TO PROVIDE ###
 #      Y     # Vector of detection status ; length: nchecklist
 #
 #      ncell     # Scalar, total number of cells
 #      nchecklist     # Scalar, total number of checklists
 #      nyear     # Vector, total number of years during SABAP1 and SABAP2; length: 2
 #
 #      adjN,weightsN,numN     # Neighborhood informations for the CAR model
 #
 #      num.knots   # Scalar, number of knots for the GAM component
 #      Hab1.basis, Hab2.basis, ...   #Matrices, bases for regression splines with climate covariates; dim: ncell * num.knots
 #
 #      layer1    # Matrix, list of cells' connected neighbors, dim: ncell*8
 #
 #      cell    # Vector indicating for each checklist the cell concerned ; length: nchecklist
 #      checklistSABAP   # Vector indicating for each checklist's SABAP ; length: nchecklist
 #      checklistyear  # Vector indicating each checklist's year within SABAP1 or SABAP2 ; length: nchecklist
 #      checklistseason  # Vector indicating for each checklist during which season it was recolted (breeding vs non-breeding) ; length: nchecklist
 #      IDobs   # Vector indicating the observer number (from 1 to NbTotObs) sampling checklist 'c' ; length: nchecklist
 #      NbTotObs    # Vector, total number of observers for each SABAP ; length: 2
 #      extra   # Vector indicating for each checklist if species detection occured during the intense birding period or not ; length: nchecklist
 #      hour.of.1st.detection   # Vector indicating for each checklist during which intense birding hour the species was detected; length:
nchecklist
 #      hour.of.intense.birding   # Vector indicating for each checklist the total number of intense birding hours ; length: nchecklist
 #
 #
 #          ### INITS TO PROVIDE ###
 #
 #      x   # Matrix for occupancy status, dim: ncell*2
 #      z     # Matrix for use status, dim: ncell*2*nyear
 #
 #      SABAP0     # Scalar, intercept for occupancy probability for SABAP1
 #      beta.Hab1.SABAP, beta.Hab2.SABAP,...  # Vectors, GAM, length: num.knots
 #      CAR.SABAP0     # Vector, spatially structured random effect for SABAP1's occupancy probability, length: ncell
 #      u   # Vector, unstructured random effect for SABAP1's occupancy probability, length: ncell
 #      tauN.S0  # Scalar, precision for spatially structured random effect for SABAP1's occupancy probability
 #      tau.u  # Scalar, precision for unstructured random effect for SABAP1's occupancy probability
 #
 #
 #
 #      persistence0, lphi1     # Scalars, intercept and slope for effect of density on persistence probability
 #      persistencerandom    # Vector, random cell effect for persistence probability, length: ncell
 #      taupersistence   # Scalar, precision for random cell effect for persistence
 #      colonization0, lgamma1     # Scalars, intercept and slope for effect of density on colonization probability
 #      colonizationrandom    # Vector, random cell effect for colonization probability, length: ncell
 #      taucolonization  # Scalar, precision for random cell effect for colonization
 #
 #      muZinittmp  # Scalar, initialization of use probabilities for the first time period for SABAP1 and SABAP2
 #      psicell, psiyear     # Matrices, random cell and year effects for exploitation probabilities, dim: 2*ncell and 2*(nyear-1) respectively
 #      taupsicell, taupsiyear  # Scalar, precision for random cell and year effect for exploitation
 #      thetacell, thetayear   # Matrices, random cell and year effects for appropriation probabilities, dim: 2*ncell and 2*(nyear-1) respectively
 #      tauthetacell, tauthetayear  # Scalar, precision for random cell and year effect for appropriation
 #
 #      pseasonSABAP1, pseasonSABAP2  # Vectors, breeding vs non-breeding season effects on detectability, length:2
```

```
#        obsSABAP1, obsSABAP2    # Vectors   ; observer random effects, length: NbTotObs
#        sigmaobs1, sigmaobs2  # Scalars, standard deviation for the observer random effects
#        CAR.obs1, CAR.obs2    # Vectors, spatially structured random effects for detection probabilities, length: ncell
#        tau.obs1, tau.obs2  # Scalars, precision for spatially structured random effects for detectability
#        delta.extra         # Scalar, variation of detection probability if species was detected outside of intense birding period
#
##############################################################


##############################################################
#                                               #
#               Level 1: Ecological process             #
#          occupancy Xi,s for cell i during SABAPs       #
#                                               #
##############################################################


############ Parameters initialization ########################

  SABAP0 ~ dnorm(0,.1)
  tauN.S0~dgamma(.1,.1)
  persistence0 ~ dnorm(0,.1)
  colonization0 ~ dnorm(0,.1)

  for(j in 1:num.knots)
  {   # Computation of GAM components
     beta.Hab1.SABAP[j]     ~ dnorm(0,2)I(-10,10)
     beta.Hab2.SABAP[j]     ~ dnorm(0,2)I(-10,10)
     beta.Hab3.SABAP[j]     ~ dnorm(0,2)I(-10,10)
     beta.Hab4.SABAP[j]     ~ dnorm(0,2)I(-10,10)
     beta.Hab5.SABAP[j]     ~ dnorm(0,2)I(-10,10)
     beta.Hab6.SABAP[j]     ~ dnorm(0,2)I(-10,10)
     beta.Hab7.SABAP[j]     ~ dnorm(0,2)I(-10,10)
     beta.Hab8.SABAP[j]     ~ dnorm(0,2)I(-10,10)

     for (i in 1:ncell)
     {
       Hab1.part.SABAP[i,j] <- beta.Hab1.SABAP[j]*(Hab1.basis[i,j])
       Hab2.part.SABAP[i,j] <- beta.Hab2.SABAP[j]*(Hab2.basis[i,j])
       Hab3.part.SABAP[i,j] <- beta.Hab3.SABAP[j]*(Hab3.basis[i,j])
       Hab4.part.SABAP[i,j] <- beta.Hab4.SABAP[j]*(Hab4.basis[i,j])
       Hab5.part.SABAP[i,j] <- beta.Hab5.SABAP[j]*(Hab5.basis[i,j])
       Hab6.part.SABAP[i,j] <- beta.Hab6.SABAP[j]*(Hab6.basis[i,j])
       Hab7.part.SABAP[i,j] <- beta.Hab7.SABAP[j]*(Hab7.basis[i,j])
       Hab8.part.SABAP[i,j] <- beta.Hab8.SABAP[j]*(Hab8.basis[i,j])
     }
   }

   for (i in 1:ncell)
   {
     Hab1.bit.SABAP[i]   <- sum(Hab1.part.SABAP[i,1:num.knots])
     Hab2.bit.SABAP[i]   <- sum(Hab2.part.SABAP[i,1:num.knots])
     Hab3.bit.SABAP[i]   <- sum(Hab3.part.SABAP[i,1:num.knots])
     Hab4.bit.SABAP[i]   <- sum(Hab4.part.SABAP[i,1:num.knots])
     Hab5.bit.SABAP[i]   <- sum(Hab5.part.SABAP[i,1:num.knots])
     Hab6.bit.SABAP[i]   <- sum(Hab6.part.SABAP[i,1:num.knots])
     Hab7.bit.SABAP[i]   <- sum(Hab7.part.SABAP[i,1:num.knots])
     Hab8.bit.SABAP[i]   <- sum(Hab8.part.SABAP[i,1:num.knots])

     u[i] ~ dnorm(0,tau.u)           # unstructured random effect

     persistencerandom[i] ~ dnorm(0,taupersistence)           # random cell effect for persistence
     colonizationrandom[i] ~ dnorm(0,taucolonization)         # random cell effect for colonization

   }
```

```
    CAR.SABAP0[1:ncell] ~ car.normal(adjN[],weightsN[],numN[],tauN.S0)  # spatially structured random effect

    tau.u ~ dgamma(0.01,0.01)
    sigma.u <- 1/sqrt(tau.u)

    taupersistence ~ dgamma(0.01,0.01)
    taucolonization ~ dgamma(0.01,0.01)
    sigmapersistence <- 1/sqrt(taupersistence)
    sigmacolonization <- 1/sqrt(taucolonization)


   # parameters used for the autologistic: #
   lphi1~dnorm(0,.1)          # "rescue effect" survival 1
   lgamma1~dnorm(0,.1)          # "rescue effect" colonization parameter 1


############ Model ########################

 for(i in 1:ncell)
 {
            ### defining occupancy for SABAP 1  ###
     x[i,1]~dbern(q[i,1])

     ### defining occupancy probability for SABAP 1###
     logit(q[i,1])<- min(100,max(-100,   qtmp[i,1]  ))
     qtmp[i,1] <- SABAP0 + Hab1.bit.SABAP[i] + Hab2.bit.SABAP[i] + Hab3.bit.SABAP[i] + Hab4.bit.SABAP[i]+ Hab5.bit.SABAP[i]+
Hab6.bit.SABAP[i] + Hab7.bit.SABAP[i] + Hab8.bit.SABAP[i] + CAR.SABAP0[i] + u[i]

     ### defining occupancy for SABAP 2  ###
     x[i,2]~dbern(q[i,2])

     ### defining occupancy probability for SABAP 2###
     logit(q[i,2])<- min(100,max(-100,   qtmp[i,2]  ))
     qtmp[i,2] <- persistence[i]*x[i,1] + colonization[i]*(1-x[i,1])


    ### defining local density###
    # ---------------------------
    #
     for (j in 1:numN[i])
     {
      ConnSite1[i,j] <- x[layer1[i,j],1]      # connected neighbors in layer 1
     }
     D[i]<- sum(ConnSite1[i,1:numN[i]])/numN[i]     #density 'rate' in first layer
    # ----------------------------


             persistence[i] <- min(100,max(-100, persistence0 + persistencerandom[i] + D[i]*lphi1))
     colonization[i] <- min(100,max(-100, colonization0 + colonizationrandom[i] + D[i]*lgamma1))

            diffx[i] <- x[i,2]-x[i,1]
 }

extent1 <- sum(x[1:ncell,1])
extent2 <- sum(x[1:ncell,2])
diffextent <- extent2-extent1

 ###############################################################
 #                                                  #
 #          Level 2: Ecological process             #
 #    use Zi,y of cell i during the yth year of SABAPs     #
 #                                                  #
 ###############################################################
```

```
 ############ Parameters initialization #######################
for (s in 1:2)
{
 muZinittmp[s]~dunif(0,1)                 # initial probability of use


 for(y in 1:(nyear[s]-1))
 {
   psiyear[s,y] ~ dnorm(0,taupsiyear)              # random year effect
   thetayear[s,y] ~ dnorm(0,tauthetayear)            # random year effect
 }

 for(i in 1:ncell)
 {
   psicell[s,i] ~ dnorm(0,taupsicell)             # random site effect
   thetacell[s,i] ~ dnorm(0,tauthetacell)           # random site effect
 }
}

taupsiyear ~ dgamma(0.01,0.01)
taupsicell ~ dgamma(0.01,0.01)
tauthetayear ~ dgamma(0.01,0.01)
tauthetacell ~ dgamma(0.01,0.01)

sigmapsiyear <- 1/sqrt(taupsiyear)
sigmapsicell <- 1/sqrt(taupsicell)
sigmathetayear <- 1/sqrt(tauthetayear)
sigmathetacell <- 1/sqrt(tauthetacell)

########### Model #######################

for (s in 1:2)
{
  for(i in 1:ncell)
  {
    z[i,s,1]~dbern(muZinit[i,s])          # use status initialization
    muZinit[i,s]<-muZinittmp[s]*x[i,s]

    for(y in 2:nyear[s])
    {
        ### defining parameters ###
        # exploitation probability
        psi[i,s,y-1]  <- min(100,max(-100, psiyear[s,y-1] + psicell[s,i] ))

        # appropriation probability
        theta[i,s,y-1] <- min(100,max(-100, thetayear[s,y-1] + thetacell[s,i] ))

        ### defining use probability  ###
       z[i,s,y]~dbern(muZ[i,s,y])          #    new use status
       muZ[i,s,y] <- muZtmp[i,s,y]* x[i,s]

       logit(muZtmp[i,s,y])<- min(100,max(-100, psi[i,s,y-1]*z[i,s,y-1]              #  exploitation if used
                    + theta[i,s,y-1]*(1-z[i,s,y-1])    ))    # appropriation  if unused)
    }
  }
}

  ############################################################
  #                                                 #
  #           Level 3 : Observation process               #
  #                                                 #
  ############################################################

 ############ Parameters initialization #######################

 delta.extra ~ dnorm(0,.1)         # variation of proba of detection after 2nd hour (case if not known whether 3h or later - indicated by 'extra[c]')
```

```
 tau.obs1~dgamma(.1,.1)
 tau.obs2~dgamma(.1,.1)

 # breeding vs non breeding season
 for (season in 1:2){
   pseasonSABAP1[season] ~ dnorm(0,.1)
   pseasonSABAP2[season] ~ dnorm(0,.1)
   }

 # observer effect
 for (l in 1:NbTotObs[1])
   {
   obsSABAP1[l] ~ dnorm(0,tauobs1)      # observer effect  SABAP1
   }

 for (l in 1:NbTotObs[2])
   {
   obsSABAP2[l] ~ dnorm(0,tauobs2)      # observer effect SABAP2
   }

 # precisions
 tauobs1 <-  1/(sigmaobs1*sigmaobs1)
 sigmaobs1 ~ dunif(0, 10)

 tauobs2 <-  1/(sigmaobs2*sigmaobs2)
 sigmaobs2 ~ dunif(0, 10)

 CAR.obs1[1:ncell] ~ car.normal(adjN[],weightsN[],numN[],tau.obs1)
 CAR.obs2[1:ncell] ~ car.normal(adjN[],weightsN[],numN[],tau.obs2)

 ############ Model ########################

   for(c in 1:nchecklist)
    {
        Y[c] ~ dbern(prob[c])             # Observations
        prob[c] <- p[c]*z[cell[c],checklistSABAP[c],checklistyear[c]]
        p[c] <- (2-checklistSABAP[c])*p.SABAP1[c] + (checklistSABAP[c]-1)*p.SABAP2[c]

        logit(p.SABAP1[c]) <- min(100,max(-100,  pseasonSABAP1[checklistseason[c]] + obsSABAP1[IDobs[c]] + CAR.obs1[cell[c]]   ))

        p.SABAP2[c] <-  (1-extra[c]) * pow((1-p.hour.SABAP2[c]),hour.of.1st.detection[c]-1) * p.hour.SABAP2[c]
                  +   extra[c]   * pow((1-p.hour.SABAP2[c]),hour.of.intense.birding[c]) * p.extra.SABAP2[c]

        IDobstmp[c] <- min(IDobs[c],NbTotObs[2])

        logit(p.hour.SABAP2[c]) <- min(100,max(-100,  pseasonSABAP2[checklistseason[c]] + obsSABAP2[IDobstmp[c]] + CAR.obs2[cell[c]]  ))
# Detection probability
        logit(p.extra.SABAP2[c]) <- min(100,max(-100,  pseasonSABAP2[checklistseason[c]] + obsSABAP2[IDobstmp[c]] + delta.extra +
CAR.obs2[cell[c]]  ))        # Detection probability
    }

}
```
